# The Production of High-Permeable and Macrovoid-Free Polysulfone Hollow Fiber Membranes and Their Utilization in CO_2_ Capture Applications via the Membrane-Assisted Gas Absorption Technique

**DOI:** 10.3390/polym17101407

**Published:** 2025-05-20

**Authors:** Pavel Țiuleanu, Artem A. Atlaskin, Kirill A. Smorodin, Sergey S. Kryuchkov, Maria E. Atlaskina, Anton N. Petukhov, Andrey V. Vorotyntsev, Nikita S. Tsivkovskiy, Alexander A. Sysoev, Ilya V. Vorotyntsev

**Affiliations:** 1 Laboratory of SMART Polymeric Materials and Technologies, Mendeleev University of Chemical Technology of Russia, 125047 Moscow, Russia; atlaskin.a.a@muctr.ru (A.A.A.); smorodin.k.a@muctr.ru (K.A.S.); kriuchkov.s.s@muctr.ru (S.S.K.); atlaskina.m.ev@muctr.ru (M.E.A.); tsivkovskii.n.s@muctr.ru (N.S.T.); shura.sys@yandex.ru (A.A.S.); vorotyntsev.i.v@muctr.ru (I.V.V.); 2 Chemical Engineering Laboratory, Lobachevsky State University of Nizhni Novgorod, 603022 Nizhny Novgorod, Russia; fox-off@mail.ru (A.N.P.); an.vorotyntsev@gmail.com (A.V.V.)

**Keywords:** membrane-assisted gas absorption, gas separation, carbon dioxide, flue gases, hollow fiber, polysulfone

## Abstract

This present study covers a complex approach to study a hybrid separation technique: membrane-assisted gas absorption for CO_2_ capture from flue gases. It includes not only the engineering aspects of the process, particularly the cell design, flow organization, and process conditions, but also a complex study of the materials. It covers the spinning of hollow fibers with specific properties that provide sufficient mass transfer for their implementation in the hybrid membrane-assisted gas absorption technique and the design of an absorbent with a new ionic liquid—bis(2-hydroxyethyl) dimethylammonium glycinate, which allows the selective capture of carbon dioxide. In addition, the obtained hollow fibers are characterized not only by single gas permeation but with regard to mixed gases, including the transfer of water vapors. A quasi-real flue gas, which consists of nitrogen, oxygen, carbon dioxide, and water vapors, is used to evaluate the separation efficiency of the proposed membrane-assisted gas absorption technique and to determine its ultimate performance in terms of the CO_2_ content in the product flow and recovery rate. As a result of this study, it is found that highly permeable fibers in combination with the obtained absorbent provide sufficient separation and their implementation is preferable compared to a selective but much less permeable membrane.

## 1. Introduction

Energy generation is one of the most important processes for human civilization. However, according to various estimates, energy generation accounts for between 25 and 85% of the emissions of the Earth’s main greenhouse gas, CO_2_ [1,2,3]. Accordingly, one of the most effective and meaningful ways to care for the Earth’s ecology is to design and develop methods to capture carbon dioxide from this source of its emissions [4].

According to various sources, over the past 100 years, the CO_2_ content in the atmosphere has continuously increased and is now up to 400 ppm, which is presumably leading to the heating of the planet’s surface [5]. If the build-up of CO_2_ continues at the current rate, by 2060, it will have passed 560 ppm, which is more than double the level of pre-industrial times [6]. The developed climate models predict that the established trend will negatively affect the global climate by 2100 [7]. As its source is anthropogenic action, nowadays, the carbon capture and storage project (CCS) is a worldwide accepted strategy that is undertaken by about 50 operational facilities today [8]. The International Energy Agency estimates that, to limit warming to below 2 °C, 2.8 Gt (billion tons) of CO_2_ per year would need to be stored by 2050 [9]. Given that a current large-scale facility captures around one million tons per year, this would suggest that over 2000 facilities need to be in service in the next thirty years.

The state-of-the-art methods with the highest prevalence are pressure swing adsorption and amine scrubbing [10,11]. Among the advantages of these methods, the main one is the high level of carbon dioxide capture (for example, short-cycle adsorption allows the capture of up to 99.9999% of the gas) [12]. However, these methods are also characterized by very significant disadvantages [13,14]. These include the regeneration of the sorbent and the complicated apparatus design. In the case of amine absorption, it is worth dwelling on the negative aspects of this process. The regeneration of absorbents is carried out by thermal desorption at 110–120 °C, which determines the high energy consumption of the process. At the same time, the regeneration is accompanied by the degradation of the sorbent due to thermal and oxidative processes. The main factor of the degradation of the amine absorbents during the extraction of CO_2_ from the flue gases is the oxidative degradation caused by a high content of O_2_ in the flue gases (up to 15 vol.%). Destruction leads to the formation of thermostable salts, which reduce the sorption capacity of the absorbent, change its rheological properties, and cause corrosion of the equipment. Additionally, it should be noted that amine absorption is also difficult to scale up and can only be adequately applied to large emission sources (as the equipment itself is quite large) [15].

A good and modern alternative to these methods is membrane gas separation [16,17,18,19,20,21]. Numerous studies devoted to membrane-based CO_2_ capture demonstrate promising results [22,23,24,25,26,27,28,29,30,31]. Anselmi et al. [22] compared three CO_2_ capture techniques, including absorption, adsorption, and membrane separation, and concluded that in terms of energy demand the membrane technique is the most preferable one. In [23], the authors proposed a novel fixed-site carrier membrane material for selective CO_2_ capture and concluded that it may be an interesting analogue to conventional solution–diffusion membranes. In [32], Kadirkhan et al. studied the CO_2_ removal process using polysulfone plasticization resistant membranes and concluded that it has the potential for further evaluation at the field stage. Another technique, which increases the separation efficiency, is the implementation of unsteady-state processes [25]. Kontos et al. studied the cellulose acetate–ionic liquid blends as gas separation membranes for enhanced CO_2_ capture performance and found that these membranes exhibit selectivity and a high potential for increased and fast CO_2_ capture [33].

The novel approach to CO_2_ capture is the appliance of hybrid techniques, which may combine the advantages of both the absorption and membrane separation techniques. Recently, Vorotyntsev et al. developed the absorbing pervaporation method [26,27]. Further, during the study of mechanisms and different cell configuration, this name was reconsidered and now it is membrane-assisted gas absorption (MAGA) [34,35,36]. This method is a combination of membrane gas separation and amine absorption. At the same time, this method uses all the advantages of its parts, while the disadvantages of amine absorption are levelled out—the sorbent is regenerated not by heating, as in the traditional method, but by desorption under the action of a pressure gradient.

However, a serious obstacle to the development of this method is the selection of the membrane materials [37]. Due to the fact that the selective part of the MAGA system can be either a membrane or an absorber, it is necessary, depending on the nature of the specific task, to select either a high-performance fiber with low selectivity or vice versa—a fiber with increased selectivity but low performance.

It is the search for such a material and a method for its manufacture that is the focus of this present work. In particular, the focus is the testing of various fiber samples obtained in the laboratory, both as independent membrane materials and as MAGA components, and the determination of the efficiency of CO_2_ capture from flue gases on MAGA modules manufactured with the use of the obtained materials.

## 2. Materials and Methods

### 2.1. Materials

The polysulfone (PSF) was chosen as a tunable and well-known material to produce hollow fibers with the desired mass transfer characteristics. Thus, we used the polysulfone UDEL P3500LCDMB7 by Solvay (Brussels, Belgium). Dimethylformamide (DMF) by Ruskhim (Moscow, Russia), tetrahydrofuran (THF) by Aldosa (Moscow, Russia), and isopropanol (IPA) by Khimmed (Moscow, Russia) were used to prepare the dope solution. Additionally, reverse osmosis water with a specific conductivity not exceeding 0.06 μS/cm was obtained on-site and hexane was purchased from Ruskhim (Russia); these were used for the fibers after the treatment procedure.

In order to obtain the absorbent, the following reagents were used. The 2-chloroethanol (99 wt%) aminoacetic acid (99%) were purchased from Sigma-Aldrich (Schnelldorf, Germany). The potassium hydroxide, ethanol, and diethyl ether (>99.5 vol.%) were kindly provided by JSC Vekos (Nizhny Novgorod, Russia). The diethyl ether and ethanol were dried using the standard procedure and stored over activated molecular sieves (4 Å) for at least 48 h. In addition, N,N-Dimethyl-2-hydroxyethylamine (99 wt%) and N,N-Bis (2-hydroxyethyl) methylamine (99 wt%) were kindly provided by «Oka-Sintez» Ltd. (Dzerzhinsk, Russia) and used without any additional purification.

The obtained membranes were characterized using single gases: nitrogen (≥99.999%), carbon dioxide (≥99.99%), and oxygen (≥99.999%), which were purchased from LLC «NII KM» (Moscow, Russia), LLC «Voessen» (Moscow, Russia), and LLC «Firma Horst» (Dzerzhinsk, Russia). After single gases permeation tests were performed, mixed gas permeation was studied with the example of the four-component mixture prepared from the same pure gases: nitrogen, oxygen, water vapors, and carbon dioxide in the proportion of 73/4.4/11/11.6 mol.%.

Among the materials mentioned above, ultrafiltration hollow fiber membranes obtained from the Laboratory of Membrane Processes of the Institute of Physical and Organic Chemistry of the National Academy of Sciences of Belarus were used to assemble the membrane-assisted gas absorption unit. Moreover, pure helium (≥99.9999%) and argon (≥99.9999%) purchased from the LLC «NII KM» (Moscow, Russia) were used for analytical purposes.

### 2.2. Asymmetric Hollow Fiber Spinning

The «dry–wet» spinning technique was used to produce the asymmetric hollow fiber membranes. To obtain a hollow fiber with high mechanical properties, the polysulfone content in the dope solution was 28 wt.%. DMF was used as a strong solvent, and its content in the dope solution was 50 wt.%. The concentration of THF as a moderate solvent was 15 wt.%, because of the positive results described elsewhere [38,39,40]. THF has high volatility and improves selective layer formation. IPA was used as a non-solvent, and its concentration was 7 wt.%. The dope composition was determined using the cloud point technique: picking the highest possible concentration of ethanol in a solution containing the PSF, DMF, and THF before the turbidity emerged (i.e., phase separation occurred).

In this way, the final dope solution composition was PSF/DMF/THF/IPA—28/50/15/7 wt.%. The preparation procedure involved several steps. The dope solution tank was filled with DMF and heated up to 55 °C. Further, PSF was loaded in small portions to prevent the polymer sticking to the stirrer anchor in the continuously stirred tank. Then, the obtained intermediate solution was thermostated for at least an hour; afterwards, the THF was added. The IPA was added to the solution after another hour of constant stirring. The final dope was stirred overnight and was then shimmered to the spinneret tank, where it was kept undisturbed for at least 8 h for solution degassing to occur. The viscosity of the dope solution was determined using the rheological complex of Anton Paar MCR-702E equipped with a cone–plate measuring system.

**Figure 1:**
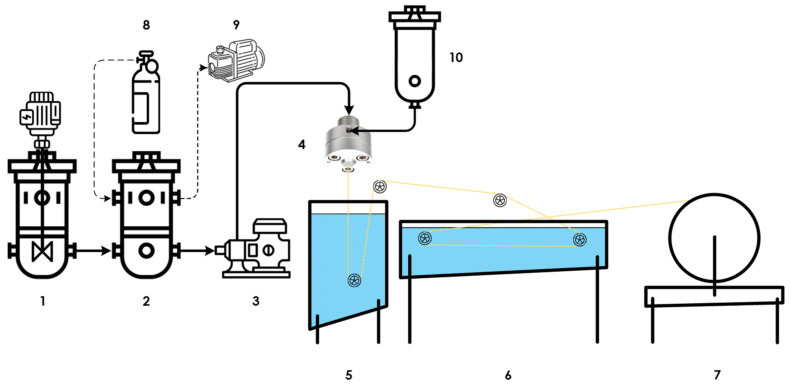
Hollow fiber spinning setup principal scheme.

The schematic diagram of the hollow fiber membrane spinning setup is given in Figure 1. Among the service equipment and sensors, the setup consisted of a dope preparation tank (1), spinneret tank (2), gear pump (3), spinneret (4), coagulation bath (5), washing bath (6), winding (7), argon cylinder (8), vacuum diaphragm pump (9), and bore tank (10).

The dope solution was prepared directly in the sealed and previously argon-purged dope tank, which is needed to prevent any contact with oxygen and moisture in the atmosphere. After the completion of the preparation procedure, the dope solution was moved to the spinneret tank, where it was kept undisturbed to remove any residual gas. Both reactors and all the pipelines were equipped with water jackets and could be heated up to 95 °C. After the dope was readied, the gear pump fed the spinneret with the dope solution on the outer orifice; meanwhile, the internal coagulant passed the spinneret through the inner one. The outer diameter (OD) of the spinnerets dope orifice was 0.5 mm and the inner diameter (ID) of the bore orifice was 0.23 mm. The formed hollow fiber then emerged into the coagulation bath, where its structure was formed and then it was washed in the bath and wound on the special wheel.

During this present study, the dope flow rate was constant and was equal to 1 g min^−1^. The water was used as an internal coagulant, and the bore flow rate was 1 g min^−1^. Both the dope and the bore temperature were constant and were 55 °C, and the water temperature in the coagulation bath was 20 °C. The winding speed was constant over the whole experiment (13 m min^−1^). The air gap between the spinneret and the water in the coagulation bath was varied from 10 to 30 cm with a step of 5 cm. This gave 5 batches of hollow fiber membrane with different structures and, consequently, with different mass transfer characteristics.

After the hollow fibers were obtained, the post-treatment procedure was applied to prevent the pore collapse and to protect the structure. Firstly, the fibers were placed in double-distilled water for 2 days; secondly, the obtained fibers were placed in IPA for 2 days; and, thirdly, they were placed in hexane for 8 h. After the procedure was completed, the fibers were dried for 12 h under vacuum at a residual pressure level of 10 kPa.

### 2.3. Hollow Fibers’ Characterization

To properly design the hybrid unit, which was the membrane-assisted gas absorption cell, all the obtained hollow fiber membranes had to be characterized not only by single gas permeance but also by taking into account the materials’ behavior with regard to the mixture to be separated. One of the main challenges in the design of the membrane-assisted gas absorption unit was choosing a suitable membrane material, taking into account its permeance, selectivity, and stability in the presence of acidic carbon dioxide, which is a plasticizer agent.

Here, the lab-scale hollow fiber membrane modules were made using 1/4″ stainless steel tube and tees provided by Swagelok (Solon, OH, USA) using epoxy resin as a potting agent at the edges of the module. Each module contained 30 fibers randomly hand-picked from each obtained bundle. Firstly, the single gas permeances were determined with regard to nitrogen, oxygen, and carbon dioxide. Secondly, the mixed gas permeance was determined based on the example of a four-component gas mixture, which consists of nitrogen, oxygen, water vapors, and carbon dioxide in the proportion of 73/4.4/11/11.6 mol.%.

**Figure 2:**
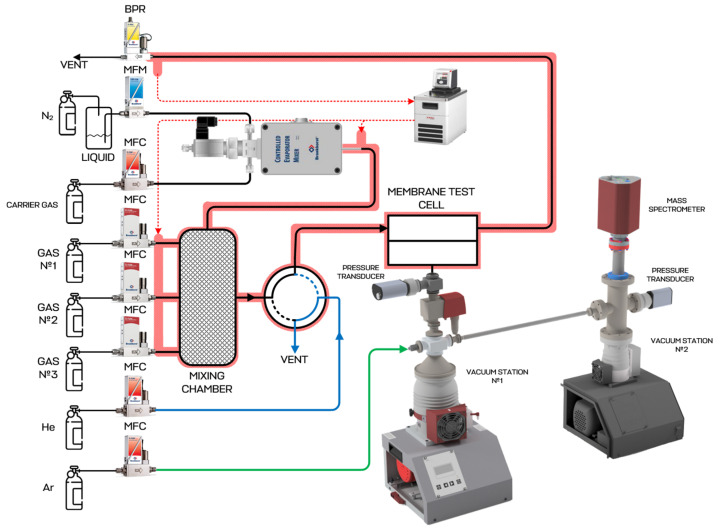
Schematic diagram of mass spectrometer-coupled experimental unit for study of membrane mass transfer properties. Black lines—gases or vapors, to be studied; blue line—purification line; green line—internal standard supply line; red lines—thermostated lines.

This study of the membrane materials was carried out on an experimental setup equipped with a quadrupole mass spectrometer. The principal diagram of the setup is presented in Figure 2. The membrane permeance test unit included a mass flow controllers FG-201 CV purchased from Bronkhorst (Nuland, The Netherlands), which was used to supply the individual gases. These controllers may be applied separately or in gas mixing mode to prepare the desired mixture into the mixing tank in real time. Another two controllers, F-201 CV and F201 CM from Bronkhorst (Nuland, The Netherlands), supplied the purge gas (helium) and internal standard (argon) into the pipelines of the test unit (in the case of the permeance of these gases not being the object of research). Helium was used in order to purge the distribution pipeline system from previously used gases and to prepare the unit before the experiment. A two-position pneumatically-actuated valve was used to connect the membrane module’s feed side with the mixing tank or helium supply pipeline. The retentate side of the membrane module was equipped with a P702 CM pressure regulator from Bronkhorst (The Netherlands) to keep the pressure level constant along the membrane.

The permeated gas entered the vacuum chamber, which was part of the pump station Hi-Cube ECO 300 from Pfeiffer (Asslar, Germany). These pumps ensured the discharge of gases from the membrane module. The pressure value in the permeate side was defined by a pressure transmitter, MPT200 from Pfeiffer (Asslar, Germany). A solenoid-operated diaphragm valve DVC 025 PX from Pfeiffer (Asslar, Germany) installed between the membrane module and pump station was used to shut down the vacuum equipment in the case of pressure surges resulting from damage to the membrane samples. Further, the permeated gas went to the QMG 250 M2 quadrupole mass spectrometer made by Pfeiffer (Asslar, Germany) connected to another pump station Pfeiffer Hi-Cube 80 (Asslar, Germany). The pressure level in the mass spectrometer chamber was monitored via a second pressure transmitter [41].

Before the experiments, the membrane module was purged using helium (at a volume flow rate of 50–150 cm^3^ min^−1^) and the mixing tank was fed with pure gases separately or multiple gases to prepare the gas mixture (at a total volume flow rate of up to 750 cm^3^ min^−1^). Argon was also supplied to the vacuum side of the gas distribution system (at a volume flow rate of 4 cm^3^ min^−1^), unless an argon permeance study was required. Helium purging was necessary to remove the air or gases remaining in the system after previous experiments. The permeation process was monitored by a mass spectrometer with the raw data refreshment rate of 1 ms. At the end of the purge procedure, the switching valve connected the mixing tank and feed side of the membrane module with an 8 ms delay. FlowPlot V.3.35 software was used to record the membrane module pressure level and all the mass flows. PV MassSpec V.21.04.01-b and PV TurboViewer V.01.04.00 were used to record the pressure level under the membrane and to obtain the mass spectra. In this way, all the raw data were collected in real time, allowing the calculation of the permeance value in every single point according to the following equations:

Permeance *Q* was determined in accordance with the following:

(1) Q=JiΔpA,

In Equation (1), *J_i_* is the volumetric flow rate of component *i*, cm^3^ min^−1^; *Δp* is the partial pressure difference across the membrane component *i*, cmHg; and the membrane area is *A*, cm^2^.

The membrane selectivity is calculated according to the following equation:

(2) α=QAQB,

The membrane selectivity is determined by a simple division of the membrane permeance values of the two gases of interest.

The software of the mass spectrometer allows the transformation of the signal of each component into the value of its partial pressure. Thus, the permeate volumetric flow rate may be estimated by the following formula:

(3) $$\frac{J_{i}}{J_{Ar}}=\frac{p_{i}}{p_{Ar}},$$

where *J_Ar_* is the volumetric flow rate of argon, cm^3^ min^−1^; *p_i_* is the partial pressure of component *i* in the permeate, cmHg; and *p_Ar_* is the partial pressure of argon in the permeate, cmHg. The error does not exceed ±2.2% of the measured value.

### 2.4. Absorbent Synthesis

A detailed description of the bis(2-hydroxyethyl) dimethylammonium glycinate ([M_2_E_2_A][Gly]) synthesis methodology is given in [42]. Briefly, equimolar amounts of ethylene chlorohydrin were added to N,N-Dimethyl-2-hydroxyethylamine to give the chloride ion compound. Then, a 10% molar excess of KOH (0.1 mol) dissolved in absolute ethanol (0.4 mol) was added to the chloride–anion compound (0.09 mol) to give the hydroxide–anion ionic compound. Next, an aqueous solution of glycine (0.1 mol) was added to the resulting ionic compound, and the mixture was stirred for 24 h at room temperature. The product was a light-yellow liquid and the yield was 90%.

**Figure 3:**
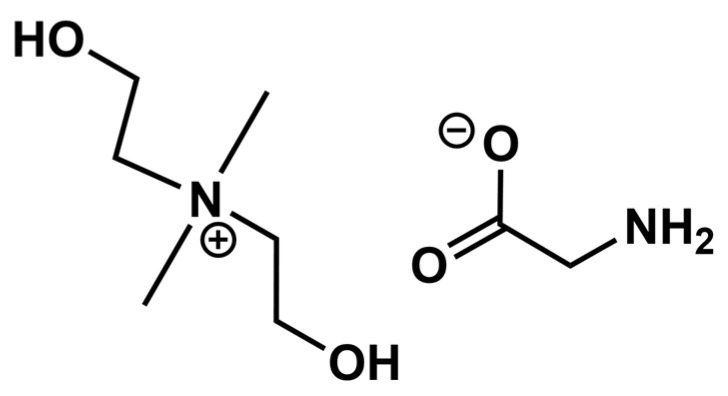
Structure of bis(2-hydroxyethyl) dimethylammonium glycinate ([M_2_E_2_A][Gly]).

The moisture content was determined using a Fischer titrator by coulometric titration. To determine the amount of moisture in the synthesized IL, a sample weighing up to 50 mg was introduced directly into the measuring module. The moisture content in IL was 0.2 wt.%. The scheme of the synthesized IL is given in Figure 3.

### 2.5. Absorbent Characterization

The sorption capacity of the solutions was determined using gravimetric analysis, using the analytical balance SHIMADZU AUW-220D (with a measurement accuracy of 1 × 10^−4^/1 × 10^−5^ g). The aqueous solutions were loaded into a glass cuvette with holes for the gas inlet and outlet. The mass fraction of MDEA or MEA in the solutions remained constant at 30 wt.%, The mass fraction of [M_2_E_2_A][Gly] in the solutions was 0, 5, 10, 20, and 30 wt.%. The cell was placed in a thermostat and maintained at a constant temperature. The experiment was carried out at atmospheric pressure. The gas flow rate was kept constant using a gas mass flow controller and was 20 cm^3^ min^−1^.

The rheological characteristics of the sorption solutions were studied on a modular compact rheometer MCR 702e MultiDrive by Anton Paar (Graz, Austria). The measurements were carried out at a temperature of 50 °C.

### 2.6. Membrane-Assisted Gas Absorption Cell Design

The membrane-assisted gas absorption technique is a pressure-driven, heat-free gas separation method that involves the selective absorption of acid impurities by a liquid absorbent with further transport to the permeate side through a polymeric membrane. In that configuration, the absorbent plays an important role in the overall unit performance.

**Figure 4:**
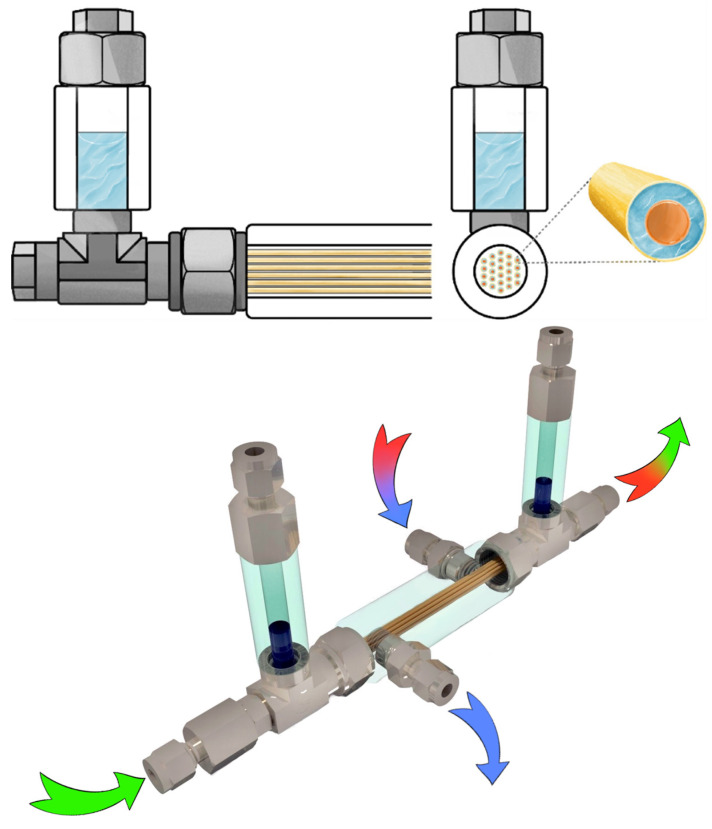
The schematic diagram of the membrane-assisted gas absorption unit.

The membrane-assisted gas absorption cell design based on hollow fiber membranes is described in detail elsewhere [36]. The key feature of this hybrid technique is the combination of absorption and membrane gas separation processes, which are simultaneously occurring in a single mass exchange unit. The configuration of the separation cell allows for the use of two types of membranes: ultrafiltration membranes and gas separation membranes, both of which are hollow fibers. The ultrafiltration fibers are of a greater diameter, so the gas separation fibers may be placed into them. The gap, formed by fibers of different diameters, is filled with liquid, which absorbs the specific gas. The schematic diagram of the cell is given in Figure 4.

**Figure 5:**
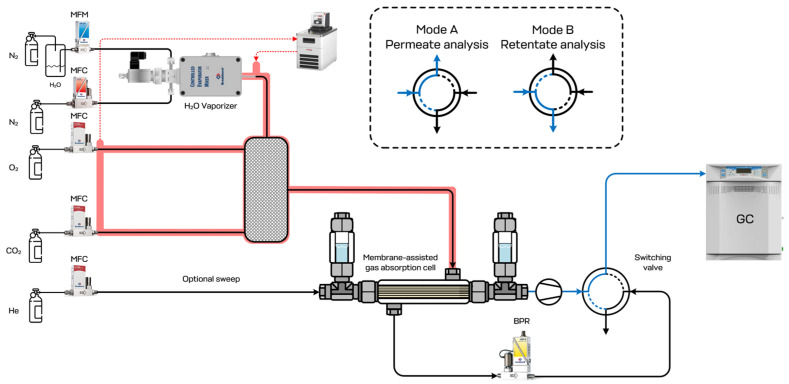
The principal scheme of the experimental setup for the evaluation of the membrane-assisted gas absorption separation efficiency. Black line—retentate line; blue line—permeate line; red line—thermostated lines.

The separation process was studied using the experimental setup, the scheme of which is given in Figure 5. During the experiment, the gas mixture, which was supplied to the membrane-assisted gas absorption cell, was prepared in real time by the dynamic mixing of 3 pure gases and water vapors. The resulting gas mixture was supplied to the feed side of the cell under a pressure of 150 kPa with a constant flow rate of 50–250 cm^3^ min^−1^ (with the nipple on the casing of the cell), moved along the combined system of fibers and liquid, and went out of the cell through the casing nipple on the opposite side of the casing. There was a back pressure regulator, P702 CM by Bronkhorst (The Netherlands), on the retentate line to maintain a constant pressure along the whole system. At the same time, the bore side of the gas separation hollow fibers were pumped with a diaphragm pump. Thus, both the permeate and retentate lines were connected to the four-port two-position switching valve. This valve was connected to the gas chromatograph GC-100 by Chromos (Dzerzhinsk, Russia) and allowed to select the gas flow to be analyzed.

**Figure 6:**
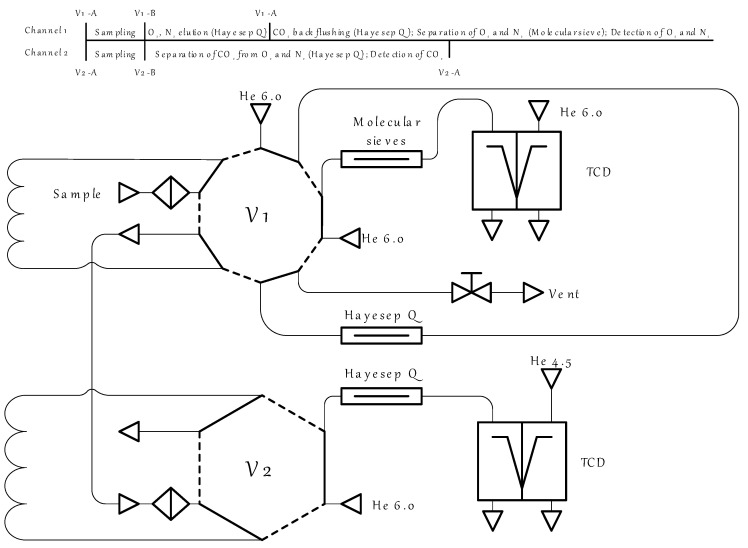
The principal scheme of the gas chromatographic system with back-flushing and detailed analysis process description.

The gas chromatograph was equipped with 2 channels, both of which were thermal conductivity detector-based, and one of them was equipped with a back-flush-to-vent system [43]. The principal scheme of the GC is shown in Figure 6. To evaluate the separation efficiency of the considered technique, it was of great importance to monitor the change in the permeate and retentate flow composition. Since the permeate stream was a highly diluted mixture of the four components (CO_2_, O_2_, N_2_, and H_2_O) in helium, to accurately determine the composition of this stream, it was necessary to determine the concentration of each of the components and then to normalize to 100 percent. In accordance with the specific task, a special two-channel gas chromatographic system was used. The GC system incorporated an electrically actuated 10-port valve (V1) to perform the sampling of the stream and the back-flushing of the CO_2_ and an electrically actuated 6-port valve (V2) for the sampling and further CO_2_ detection. Both valves were thermostatic. Helium of a high purity was used as a gas carrier. Two types of chromatographic columns were used: the Hayesep Q to separate the permanent gases from CO_2_ and the 13X molecular sieve column, which served to separate O_2_ and N_2_. Initially, both valves were in the A position (solid line), and the sample flowed through the V1 sampling loop and moved to the V2, filling the sampling loop. On the next step, both valves were switched to position B (the dash–dot line), and the permanent gases were separated from CO_2_ in GC channel 1; meanwhile, the sample from the V2 valve was moved to the Hayesep Q chromatographic column, where the separation of CO_2_ from O_2_ and N_2_ occurred in isothermal conditions and was detected using TCD. After the permanent gases fraction was eluted but before the CO_2_ elution, V1 was switched back to reverse the helium flow and back-flush the CO_2_ adsorbed on the Hayesep Q column. Meanwhile, the O_2_ and N_2_ were separated using the 13X molecular sieve column and detected using TCD.

It is also possible to evaluate the mixed gas permeance through the combined membrane-absorbent system based on the results of the GC analysis of the permeate flow during this process study. To determine the permeance of each component, the vacuum pump-generated flow was replaced with a helium sweep according to Equation (3) from [44]:

(4) $$Q_{A}=\frac{x_{A}^{P}}{x_{He}^{P}A∆p_{A}},$$

where *Q_A_* is the permeance of component *A*, *S* is the helium sweep gas flow rate, $x_{A}^{P}\;$ is the mole fraction of component *A* in the permeate flow, $x_{He}^{P}\;$ is the mole fraction of helium in the permeate flow, *A* is the area of the membrane, and *p_A_* is the partial pressure difference across the membrane for component *A*.

## 3. Results and Discussion

### 3.1. Hollow Fiber Membranes’ Mass Transfer Properties

First, the obtained PSF hollow fiber membranes were characterized by their single gas permeances with regard to nitrogen, oxygen, and carbon dioxide. The results are shown in Table 1, where the mass transfer properties of the five samples are given with the air gap used in the spinning process.

As is seen from the results shown in Table 1, there are pin holes in samples #1–3, as the measured selectivity of the obtained PSF fibers are much lower than their intrinsic value [45,46,47]. As all the fibers were obtained at the same temperature of 55 °C and the same take-up speed, here only the air gap size value affects the mass transfer characteristics of the spun samples. The formation of a very thin selective layer or its formation with many defects is connected with the time spent in the air gap, where highly volatile THF readily evaporates. It is seen that, in the case of this specific dope composition and spinning technological parameters, the air gap size of 25–30 cm allows the formation of a sufficiently thin and defect-free selective layer. In accordance with Knudsen diffusion and the Hagen–Poiseuille model, the membrane selectivity of the oxygen–nitrogen pair equals the square root of the inverse ratio of their molecular weights: α_KN_(O_2_/N_2_) = 0.935; and the ratio of oxygen to nitrogen permeance is equal to the inverse ratio of the gas viscosities: approximately 0.867. For sample #5 of this present study, the apparent thickness of the selective layer is about 53 nm.

**Figure 7:**
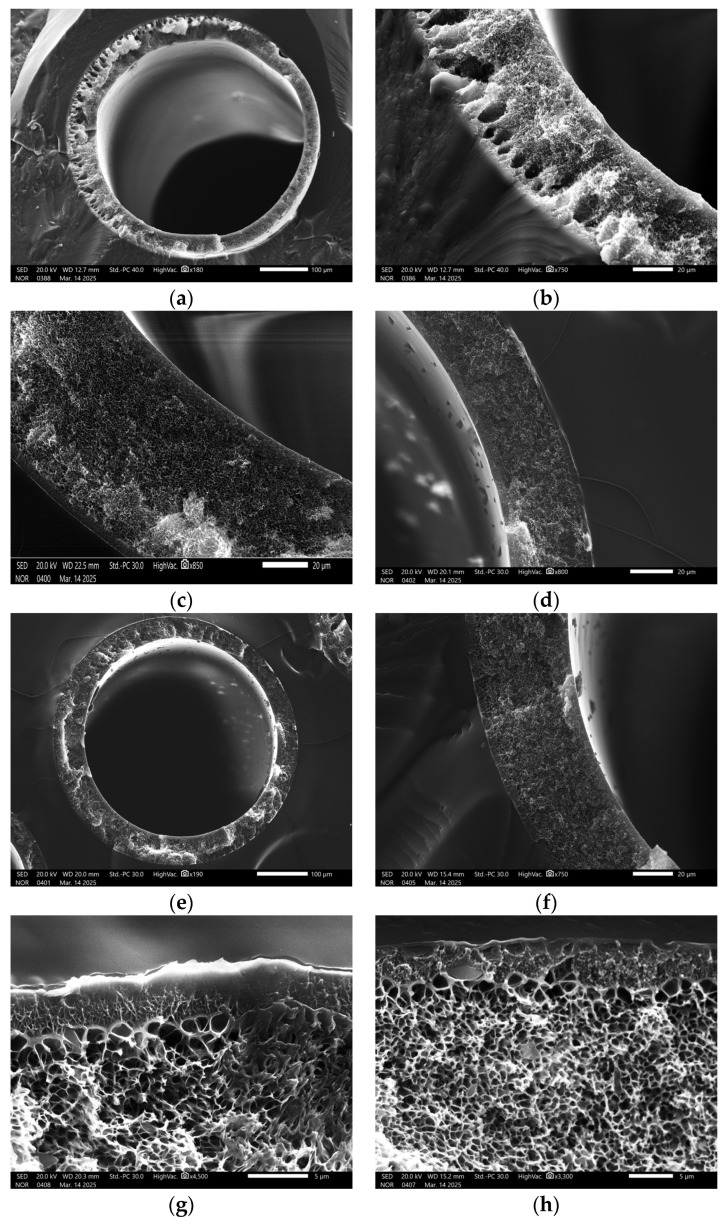
SEM microphotographs of obtained PSF hollow fibers. (**a**,**b**)—cross-section images of sample #1; (**c**–**f**)—cross-section images of samples #2, #3, #4, and #5, respectively; and (**g**,**h**)—zoomed in cross-section image of surface region of samples #4 and #5, respectively.

As is seen from the SEM microphotographs given in Figure 7, there are macrovoids with a length of up to 27 μm and a width of up to 16 μm at the lumen side of the sample #1 (a–b) observed at the cross-section. Meanwhile, the cross-section images of samples #2–#5 (c–f) have a different structure and are macrovoid-free. The (g) and (h) zoomed-in cross-section images of samples #4 and #5, respectively, show the absence of any voids in the selective layer region of 4 μm, composed of a dense skin layer, which is supported by a sponge-like structure of many micropores and microchannels.

The macrovoid formation on the lumen side of the fibers is occurring during the non-solvent induced phase separation (NIPS) process. A rapid exchange of the solvent (DMF and THF) and non-solvent (water) takes place across the interfacial layer at the moment of the contact of the dope solution and the internal coagulant. Due to the diffusion-driven movement of the coagulation front through the fiber wall, there is the nucleation of the polymer-lean phase occurring on the lumen side [38,48]. Once initiated, nuclei can expand isotopically unless the rigidity of the nucleus wall counteracts the diffusive forces driving their growth. Macrovoids form when the inward diffusion rate of water significantly surpasses the outward diffusion rate of the solvent from the nucleus [49]. Taking into account the difference in the diffusion rates of the coagulant (water) and larger organic solvent molecules, it is likely that this effect is responsible for the macrovoid formation. Moreover, the diffusion influx of the solvent from the dope into the nucleus through the plasticized “front side” is greater than the outflow of the solvent from the nucleus through the vitrified “backside”. Consequently, this solvent movement may determine the shape and the size of the macrovoids in the fibers.

This phenomenon is likely responsible for the macrovoid formation, considering the substantial difference in diffusion coefficients between the water and larger organic molecules such as DMF and THF. Additionally, the influx of the solvent from the dope into the nucleus through the plasticized “front side” exceeds the outflow of the solvent from the nucleus through the vitrified “backside”. This solvent ingress is thought to determine the size and teardrop shape of the observed macrovoids.

After the single gas permeation test, the mixed gas permeation was studied. The results are given in Table 2. As is seen from the results shown in Table 2, the selectivity of all samples decreases in the comparison with single gas permeation. At the same time, the membranes’ permeance to N_2_ remains almost unchanged; meanwhile, the transfer of oxygen and carbon dioxide is lowered in the case of mixed gas permeation. As the partial pressure ratio across the membrane is lower in the case of the gas mixture, compared to single gases, especially in the case of O_2_ and CO_2_ (4.4 and 11.6 mol.%), it is natural that membrane permeation is lower with regard to the components of the gas mixture. At the same time, the main component of the mixture is nitrogen (73 mol.%) and, as a result, the fibers’ permeance with regard to nitrogen differs slightly or does not differ at all. In addition to nitrogen, oxygen, and carbon dioxide, the water vapor transport was studied. As it is seen, the hollow fibers’ permeance to water vapors is at least 2.4 times higher than its CO_2_ value. At the same time, a decrease in the air gap and the compaction of the internal structure of the fiber decreases the water vapor mass transfer. Nevertheless, it remains the highest among those observed and equals 715.22 GPU for sample #5, forming H_2_O/CO_2_ selectivity of 10.2.

To put the results of this present study in the context of polysulfone hollow fiber state-of-the-art production, it is of interest to consider different dope solutions and spinning process parameters. Some data from the literature were systemized and are presented in Table 3. It is worth noting that, among the compared PSF fibers, there are two groups: untreated polysulfone and silicone-coated fibers (usually the polydimethylsiloxane is used to cure the pin-holed surface). Among the untreated fibers, the obtained membranes have a higher permeance and selectivity in comparison with the results given in [39,50]. Moreover, in [50], a viscous dope solution was used (with a polymer content of 37 wt.%). In practice, it is difficult to maintain the consistency of the characteristics of the resulting fiber, and it can be the potential source of fiber breakages during the spinning process. Additionally, propionic acid used as a non-solvent is quite a dangerous medium. Among the treated membranes described in [38,45], the permeance of oxygen is comparable with the value of the studied fibers, but its selectivity is higher (6 and 6.4 versus 5.4). Its carbon dioxide permeance is almost 1.6 times higher, resulting in a high CO_2_/N_2_ selectivity of 33. Nevertheless, the PDMS post-treatment process includes several additional steps, such as solution preparation, fiber treatment, drying, and fixing the applied layer. Consequently, it increases the hollow fiber membrane production costs and may be the additional source of defects.

In addition to the hollow fibers made of polysulfone, Ying Li et al. reported the modification of PSF films via Polyvinylalcohol in order to increase the CO_2_/N_2_ separation performance [51]. The modification of PSF films by PVA leads to a decrease in permeance of up to ~1.8–3.3 and an increase in selectivity of up to 78; meanwhile, a pure PSF film provides a CO_2_ permeance of 6 GPU and a CO_2_/N_2_ selectivity of 37. Obviously, the prepared films cannot compete with hollow fibers in terms of permeance due to the thickness of the selective layer, but it may be considered as a prospective way to enhance the PSF membranes’ performance.

### 3.2. Absorbent Properties

Ionic liquids are promising compounds for acid gas capture applications and have a wide range of positive properties, namely, a high absorption capacity for a wide range of inorganic and polar organic compounds, low vapor pressure, and relatively high chemical and thermal stability. Since it is currently very important to pay attention to the environmental friendliness of the components used, it is necessary to design and use non-toxic «green» materials, for example, those based on amino acids. In this regard, an ionic liquid with a choline-based cation and amino acid anion was used in this work, which has shown its potential both as an individual CO_2_ absorbent and as an effective additive to amino alcohol solutions.

Aqueous solutions of MDEA and MDEA with different contents of ionic liquid were prepared in order to determine the effect of the solution composition on the carbon dioxide absorption, before choosing the most efficient one. The mass fraction of alkanolamine in all the solutions was 30 wt.%. The absorbing properties of the solutions with a mass fraction of IL in the range of 5–30 wt.% were determined using the gravimetric technique. The constant carbon dioxide flow was equal to 20 cm^3^ min^−1^. The test cell was thermostated, and its temperature was 40 °C. The cell was weighed at 5 min intervals during the first 10 min of sorption, then at 10 min intervals up to the 40th minute of the process, and then at 20 min intervals up to the 80th minute of the absorption process. The indicator of the end of the sorption process was a decrease in the cell mass over several measurements (the evaporation of the solution). At the same time, the same experiment was conducted with a non-sorbed gas, nitrogen, to account for the amount of solution evaporating during the gas bubbling.

**Figure 8:**
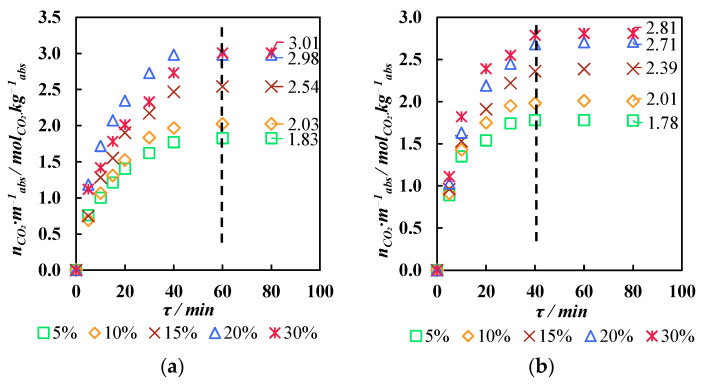
Kinetics of carbon dioxide saturation of solutions with different IL contents: (**a**)—MEA-based solutions; and (**b**)—MDEA-based solutions.

**Figure 9:**
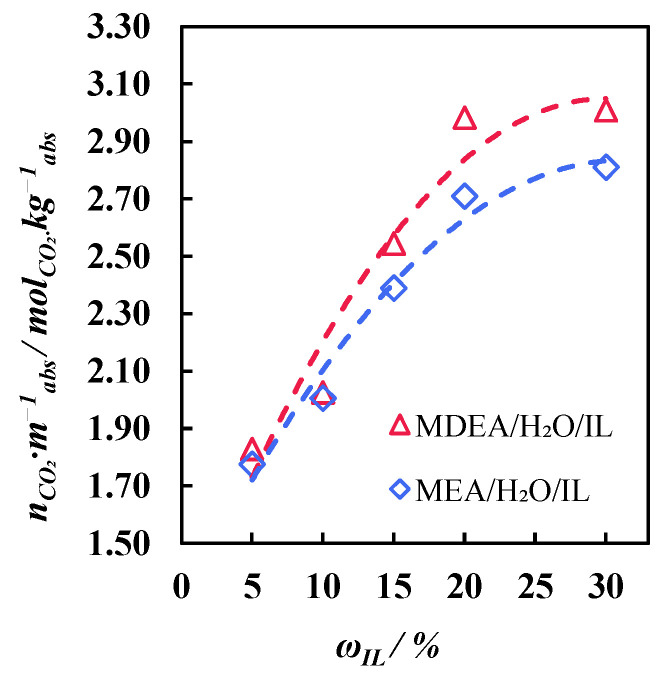
Dependence of the sorption capacity of solutions on the mass content of IL. Dotted lines are the trends of sorption capacity change over the mass content of IL.

The kinetics of the carbon dioxide absorption process by the prepared solutions is shown in Figure 8 (a—the MEA-based solution, and b—the MDEA-based solution). With an increase in the content of the ionic liquid in both the MEA-based and MDEA-based solutions, the sorption capacity of the absorbent increased. However, saturation occurred more slowly. Thus, for the solutions with MEA and MDEA, the maximum sorption capacity was 1.78 and 1.84 ${mol}_{{CO}_{2}}{kg}^{abs}$ (5% IL), 2.01 и 2.03 ${mol}_{{CO}_{2}}{kg}^{abs}$ (10% IL), 2.39 и 2.54 ${mol}_{{CO}_{2}}{kg}^{abs}$ (15% IL), 2.71 и 2.98 ${mol}_{{CO}_{2}}{kg}^{abs}$ (20% IL), and 2.81 и 3.01 ${mol}_{{CO}_{2}}{kg}^{abs}$ (30% IL), respectively. As is seen, the values of the maximum sorption capacity of all the MDEA-based solutions, although insignificant in some experiments, are still higher than with MEA. In addition, reaching a plateau (achieving saturation) for the MDEA-based solutions occurred at approximately the 60th minute of the process (except for the solution containing 30% IL). And for the MEA-based solutions, saturation was achieved only after 40 min. The highest sorption capacity is demonstrated by a solution based on MDEA with a 30% IL content (3.01 ${mol}_{{CO}_{2}}{kg}^{abs}$ ); however, this is only 1% higher than for a solution with 20% IL (2.98 ${mol}_{{CO}_{2}}{kg}^{abs}$ ). Moreover, this value is higher than for the MEA solution containing 30% IL, but only 9.6%. Taking into account the results of the absorption and viscosity studies, it is preferrable to use an MEA-based 30 wt.% IL solution to separate the flue gases in a combined membrane-assisted gas absorption system. Taking into account the results of the absorption process kinetic study and comparing the absorption capacities (Figure 9), it seems more preferable to use an MEA-based 30 wt.% IL solution to separate the flue gases in a combined membrane-assisted gas absorption system.

As is known from the data from the literature, nitrogen and oxygen hardly dissolve in aqueous MEA or MDEA solutions [52,53]. Thus, the appliance of proposed solutions with IL should provide high selectivity for the membrane-assisted gas absorption process. The results and discussion of the study of flue gases separation is given below.

### 3.3. Membrane-Assisted Gas Absorption Performance

After the complex study of membrane material and absorbent solutions, it was decided to apply the MEA-based 30 wt.% IL absorbent and two types of the produced hollow fibers: the high-permeable non-selective sample #1 with macrovoids in its structure and the low-permeable sample #5 with moderate selectivity, which is common for uncured PSF membranes. The main goal was to determine the optimal combination of a hybrid membrane-absorbent system to provide not only the efficient concentration of carbon dioxide but to also perform its capture, in terms of a high recovery rate.

During this experimental study, the feed flow was varied on a range of 50–250 cm^3^ min^−1^ to observe the system performance and determine the ultimate efficiency for each configuration. It is obvious that the system with a low-permeable membrane does not allow processing at the same flow rate as the system with a membrane that is almost 15 times more permeable. Nevertheless, the selective sample may be used at a lower stage cut, achieving a higher CO_2_ concentration in the permeate flow. In this way, the differences in the permeate composition and overall performance between the different systems should be studied to choose one that is appropriate for a specific separation process.

**Figure 10:**
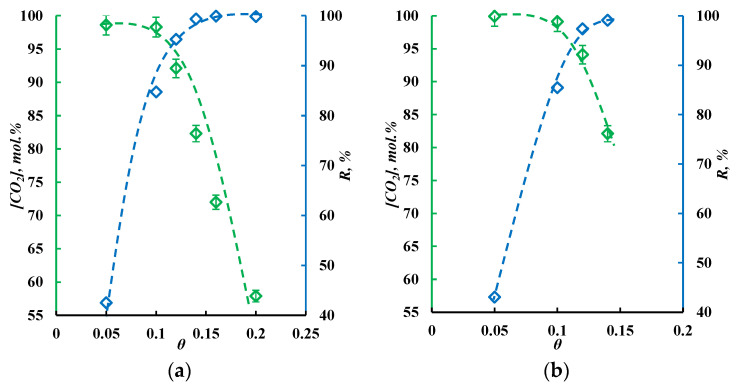
The dependencies of CO_2_ concentration in the permeate flow and its recovery rate on the stage-cut value. (**a**)—fiber sample #1 with an MEA-based aqueous solution with 30 wt.% of IL with a feed flow rate of 250 cm^3^ min^−1^; and (**b**)—fiber sample #5 with an MEA-based aqueous solution with 30 wt.% of IL with a feed flow rate of 75 cm^3^ min^−1^. Dotted lines are trends of CO_2_ and recovery rate change over the stage-cut value.

The results presented in Figure 9 contain the results of the study of the membrane-assisted gas absorption process during the separation of flue gases. Figure 10a illustrates the dependencies of the CO_2_ concentration in the permeate flow and its recovery rate versus the stage cut obtained for the system with fiber sample #1 and an MEA-based aqueous solution with 30 wt.% IL. Figure 10b shows graphs obtained for the same solution placed in a gap between the UF hollow fibers and sample #5.

As is seen from the graphs in Figure 10a, using sample #1 of the obtained fibers allows the separation of the flue gases at a feed flow rate of 250 cm^3^ min^−1^ in a quite wide range of stage cut values (0.05–0.2). The lowest value of the stage cut of 0.05 provides the permeate stream, which completely consists of CO_2_. Nevertheless, those process conditions do not meet the requirements of a CO_2_ recovery rate of more than 90%. Under a stage cut of 0.05, its value equals 42.5%. The common requirements of flue gas processing are a capture of more than 90% of CO_2_ with its content in the product flow being not lower than 95 mol.%. In these terms, the membrane-assisted gas absorption technique allows the efficient capture of the CO_2_ under stage cut values between 0.1 and –0.15. Further increases in the stage cut increase the recovery rate but rapidly drop the CO_2_ content in the permeate stream. Thus, the CO_2_ concentration in the permeate is always lower than 95 mol.% and reaches only 57.9 mol.% at a stage cut of 0.2. Meanwhile, the recovery rate is close to 99% at stage cut values in the 0.14–0.2 region.

As is seen from the graphs in Figure 10b, the application of the obtained hollow fiber sample #5 meets the requirements of CO_2_ capture between stage cut values of 0.1 and 0.15. It is worth noting that the feed flow is 75 cm^3^ min^−1^, due to the much lower permeance of the used hollow fibers. Moreover, it does not provide the ability to perform the separation under higher stage cut values. Meanwhile, the CO_2_ content in the permeate is higher in the whole observed range of stage cuts compared to the process performed on fiber sample #1. Thus, the difference in CO_2_ concentration in the permeate stream varies from 0.8 to 2 mol.%.

Both sample #1 and sample #5 among the obtained hollow fiber PSF membranes efficiently capture CO_2_ from flue gases via the membrane-assisted gas absorption process, but the more permeable fibers are more preferable due to their much higher productivity. The selectivity of the membrane itself does not make a defining contribution in overall process selectivity. As was expected, the absorbent plays a key role, providing extremely high selectivity, which provides the possibility of producing a product flow rate with the required CO_2_ concentration capturing more than 90% of the incoming carbon dioxide. Compared to the classic membrane technology, multi-stage or multi-step technological schemes are needed to efficiently capture CO_2_ [54,55]. The membrane-assisted gas absorption technique may provide the separation in one stage and requires only one vacuum pump on the permeate side instead of numerous stages and/or steps with a pre-compressed feed.

As was mentioned in Section 2.6, it is possible to approximately evaluate the permeance of each component through the combined membrane-absorbent system. It is recommended to measure that value under the stage cut of 0.01 [44]. As the lowest achievable stage cut value during that study was 0.05, the permeance values were determined under that condition. In that particular case, the approximate permeance of the system was 105, 0.2, and 1.1 GPU with respect to CO_2_, N_2_, and O_2_, respectively, which results in the selectivity of 525 and 95.5 for the CO_2_/N_2_ and CO_2_/O_2_ pairs. Comparing these values with the characteristics of the initial membrane #1, it is possible to conclude that, despite the drop in permeance of up to almost 10 times, the selectivity rise is almost 160 times. This is due to the solution absorption properties, which interact only with CO_2_; meanwhile, the nitrogen and oxygen cannot pass through the membrane-absorbent system and are removed as a retentate flow. Regarding the water vapors, it is hard to evaluate the system permeance as the absorbent accumulates them, and this phenomenon affects the mass transfer properties of the system.

Nevertheless, there are several problems, which should be solved prior to considering membrane-assisted gas absorption as the most significant technology. One of them is water vapors dissolving in the amine aqueous solutions, which is under long-term continuous utilization, leading to a decrease in the alkanolamine and IL content due to the accumulation of the incoming water in the solution. During this study, it was observed that the efficiency of the process, which is performing in a steady-state mode, decreases after 24–27 h due to the dilution of the absorbent. A possible solution is the usage of an additional gas dryer unit, such as the glycol gas dehydration system, prior to the membrane-assisted gas absorption cell.

Another problem is the complex assembling of the membrane-assisted separation cell, which in lab-scale conditions involves the placing of gas separation fibers into UF fibers of a larger diameter. This complex step involving hand-made assembling may be replaced by the simultaneous spinning of two types of fiber with different diameters and structures on a special custom-made spinneret.

## 4. Conclusions

As a result of this complex membrane-assisted gas absorption process study, it was found that the process is suitable for capturing CO_2_ from flue gases. Here, a combination of new materials was studied with regard to the specific features of the studied process. In this present work, five samples of hollow fiber gas separation membranes obtained from PSF/DMF/THF/IPA dope solutions were studied comprehensively.

The mass transfer of quasi-real flue gases with the inclusion of water vapors was in the spotlight of this paper, in contrast to a study of single gas permeation. The SEM microphotographs indicate that it is possible to spin macrovoid-free fibers with a high permeance that are close to the intrinsic selectivity of polysulfone. On the other hand, the absorbent solution was studied with regard to its CO_2_ absorption capacity and process kinetics. Based on these results, the optimal composition of the absorbent solution was chosen—an MEA-based aqueous solution with 30 wt.% of IL.

This membrane-assisted gas absorption process study, implemented on two fiber samples (with a high permeance but low selectivity and vice versa), demonstrates that it is possible to utilize the investigated technique to capture CO_2_ from flue gases. Comparing these two combinations of materials, it was found that in both cases the separation goals (a recovery rate of >90% of CO_2_ and its content in the product flow being >95 mol.%) may be achieved, but a higher productivity of the process is provided by the high-permeable sample #1 of the obtained fibers.

## Figures and Tables

**Table 1:** Mass transfer characteristics with regard to the single gases of the hollow fiber membrane samples, obtained at different air gaps.

№	Air Gap, cm	Q, GPU			α	
		N_2_	O_2_	CO_2_	CO_2_/N_2_	O_2_/N_2_
1	10	316.49	411.44	1171.01	3.7	1.30
2	15	214.66	364.93	1030.37	4.8	1.70
3	20	123.9	284.96	805.35	6.5	2.3
4	25	34.84	108.00	463.37	13.3	3.10
5	30	3.88	20.96	75.27	19.4	5.40

@ 150 kPa and 25 °C; 1 GPU = 1 × 10^−6^ cm^3^ cm^−2^ s^−1^ cmHg^−1^.

**Table 2:** The mass transfer characteristics with regard to the mixed gases of the hollow fiber membrane samples, obtained at different air gaps.

№	Air Gap, cm	Q, GPU				α		
		N_2_	O_2_	CO_2_	H_2_O	CO_2_/N_2_	O_2_/N_2_	H_2_O/CO_2_
1	10	317.27	349.00	1046.99	2512.78	3.3	1.10	2.4
2	15	214.48	311.00	900.82	2882.62	4.2	1.45	3.2
3	20	122.54	257.34	747.49	4036.45	6.1	2.1	5.4
4	25	34.85	107.00	442.6	3300.00	12.7	3.07	7.5
5	30	3.73	19.40	70.12	715.22	18.8	5.20	10.2

@ 150 kPa and 25 °C; 1 GPU = 1 × 10^−6^ cm^3^ cm^−2^ s^−1^ cmHg^−1^.

**Table 3:** A comparison of the mass transfer properties of the PSF hollow fibers obtained in this present study with data from the literature.

Dope Composition	Q, GPU		α		Ref.
	O_2_	CO_2_	O_2_/N_2_	CO_2_/N_2_	
PSF/DMF/THF/IPA	20.96	75.27	5.4	19.4	Present study
PSF/DMA/THF/EtOH *	25.6	-	6	-	[38]
PSF/NMP/THF/EtOH/PEG		17.6	-	-	[39]
PSF/NMP/H2O/EtOH *	23.2	119.1	6.4	33	[45]
PSF/NMP/PA	20		4.4		[50]

* PDMS-treated fibers.

## Data Availability

The original contributions presented in this study are included in the article. Further inquiries can be directed to the corresponding author.
